# Antibacterial activity of crocin-loaded niosomes against foodborne pathogens isolated from cream pastries

**DOI:** 10.1186/s12866-026-05059-8

**Published:** 2026-04-25

**Authors:** Ebrahim Molaee-aghaee, Ramin Akbari Dehcheshmeh, Razieh Noroozi, Mohammad Reza Pourmand, Parsa Abassi, Masoomeh Amini, Samira Karimaei

**Affiliations:** 1 Division of Food Safety and Hygiene, Department of Environmental Health Engineering, School of Public Health, Tehran University of Medical Sciences, Tehran, Iran; 2 Department of Food Science and Technology, School of Nutritional Sciences and Dietetics, Tehran University of Medical Sciences, Tehran, Iran; 3 Department of Pathobiology, School of Public Health, Tehran University of Medical Sciences, Tehran, Iran; 4 Department of Medical Nanotechnology, School of Advanced Technologies in Medicine, Tehran University of Medical Sciences, Tehran, Iran

**Keywords:** Antibacterial Activity, Cream Pastries, Crocin, Niosomes, Encapsulation Efficiency, Foodborne Pathogens

## Abstract

**Background:**

Cream pastries, due to their high moisture and nutrient content, are susceptible to growth of foodborne pathogens, which endanger human health. Crocin, an apocarotenoid glycoside from saffron (*Crocus sativus*), exhibits broad-spectrum antimicrobial activity. This study aimed to isolate food-derived strains of common foodborne bacteria, including *Staphylococcus aureus*, *Escherichia coli*, and *Bacillus cereus*, from cream pastries collected in Iran, and to comparatively evaluate the in vitro antibacterial activity of crocin in free and niosome-encapsulated forms.

**Results:**

A total of 178 cream pastry samples were analyzed for microbial contamination. Target bacteria were isolated, cultured, and identified by standard routine microbiological techniques. Crocin-loaded niosomes prepared by the thin-film hydration method formed nano-sized vesicles (139 ± 0.7 nm), with a morphology, surface charge (zeta potential: +9.9 ± 0.5 mV), and a high encapsulation efficiency (72 ± 0.5%) and a sustained in vitro release profile over 48 h (up to 53% cumulative release at pH 6.8). The antibacterial activity of free and encapsulated crocin against isolated pathogens was assessed by measuring the minimum inhibitory concentration (MIC) and minimum bactericidal concentration (MBC), which were treated as discrete, stepwise endpoints and reported descriptively. *S. aureus* was detected in 2 (1.1%), *E. coli* in 1 (0.56%), and *B. cereus* in 1 (0.56%) of samples. Niosomal crocin exhibited lower MIC and MBC values against *S. aureus* and *E. coli* compared to free crocin, corresponding to a two-fold reduction in MIC. In contrast, no difference in MIC or MBC values was observed for *B. cereus*.

**Conclusions:**

Niosomal encapsulation enhances the physicochemical stability of crocin and improves its antibacterial performance against Gram-positive and Gram-negative foodborne pathogens, offering a biocompatible, natural antimicrobial strategy for improving microbial safety of perishable foods such as cream pastries. Future studies should investigate the underlying mechanisms, such as biofilm disruption and membrane permeabilization, and evaluate the efficacy in real food matrices.

**Supplementary Information:**

The online version contains supplementary material available at 10.1186/s12866-026-05059-8.

## Introduction

Foodborne diseases remain a major global health concern, with millions of cases reported annually due to the consumption of food contaminated with pathogenic microorganisms [1]. The most common agents are *Staphylococcus aureus*,* Escherichia coli*, and *Bacillus cereus*. These bacteria pose a multifaceted challenge because they produce thermostable enterotoxins that can withstand standard food processing temperatures and form biofilms on food contact surfaces and within food matrices [2]. The biofilm formation enhances their ability to survive environmental stresses, making them more resistant to sanitization and traditional preservation methods, which complicates control efforts. This microbial resilience is especially problematic in nutrient-rich, high-moisture foods, such as cream-filled pastries, which provide ideal environments for rapid microbial growth and toxin excretion. Such conditions both promote quick microbial proliferation and increase the risk of toxin production, raising the likelihood and severity of foodborne outbreaks. Furthermore, the rise of resistance to common food-grade antimicrobials and preservatives has reduced the effectiveness of current food-grade antimicrobial agents, prompting a global demand for safer, natural, and sustainable solutions [3, 4].

Over the recent years, plant-based and natural antimicrobial compounds have been the focus of many studies aimed at increasing food shelf life and safety. Plant secondary metabolites are increasingly recognized for their antimicrobial activities, combined with favorable safety and biocompatibility profiles, making them attractive candidates for integration into food preservation systems. Among these, phytochemical secondary metabolites, including phenolic compounds, terpenoids, carotenoids, gum-derived ingredients, and essential oils, have demonstrated broad-spectrum antimicrobial efficacy and safety in food systems [5–7].

Among bioactive carotenoids, crocin, a water-soluble apocarotenoid glycoside derived from the stigmas of *Crocus sativus* L. (saffron), has been used in many researches. Extensive pharmacological studies have validated the diverse biological activities of crocin, including antioxidant, anti-inflammatory, neuroprotective, and antimicrobial capacities. Its inhibition of both Gram-positive and Gram-negative foodborne pathogens underscores its potential as a natural preservative for food safety [8–10].

Pharmacological studies confirm crocin’s broad biological activities, including antioxidant, anti-inflammatory, neuroprotective, and antimicrobial effects. Its inhibition of both Gram-positive and Gram-negative foodborne pathogens underscores its potential as a natural preservative for food safety.

However, while crocin is an effective antimicrobial agent, it has many physicochemical limitations that restrict its use in food applications. Environmental factors such as exposure to light, heat, oxygen, and moisture critically impact crocin’s stability and efficacy. Furthermore, crocin’s low lipid solubility and limited bioavailability restrict its dispersion and antimicrobial effectiveness in complex food matrices [11, 12]. To overcome these challenges, nano-encapsulation technologies have emerged as advanced delivery systems, enhancing the stability, bioavailability, and controlled release of bioactive compounds. Niosomes- vesicles composed of nonionic surfactants and cholesterol- can encapsulate hydrophilic and lipophilic substances, protecting sensitive compounds from degradation, improving stability under adverse environmental conditions, enabling controlled release profiles, and potentiating antimicrobial activity, thus addressing the limitations of traditional delivery methods [13, 14]. Given the high probability of contamination of cream pastries with pathogenic microorganisms such as *S. aureus*,* E. coli*, and *B. cereus*, and the high popularity and widespread consumption, adopting an innovative and targeted antimicrobial approach is essential. This study proposes a novel antimicrobial strategy by synthesizing and characterizing crocin-encapsulated niosomes, aiming to compare their antibacterial efficacy against major foodborne pathogens with that of free crocin. While niosomal encapsulation is an established approach, its application for crocin in the context of food-derived pathogens remains underexplored, and the specific efficacy of crocin-loaded niosomes against strains directly isolated from cream pastries has not been investigated. Moreover, the stability and release of crocin in food-relevant conditions, such as neutral pH and high moisture, have not been systematically assessed. These unresolved issues constitute the critical knowledge gap addressed in the present study. Crocin-loaded nanocarriers have been suggested to enhance antibacterial efficacy through mechanisms such as improved membrane interaction and potential biofilm disruption; however, investigation of these mechanisms and validation in food matrices require dedicated, application-oriented studies.

By enhancing the antimicrobial properties and stability of crocin through niosomal encapsulation, this approach aims to provide a natural, effective preservative alternative to improve the microbial safety of high-risk confectionery products.

Therefore, this study aims to address this gap by developing crocin-loaded niosomes and rigorously evaluating their antibacterial efficacy against contemporary food-derived isolates of *Staphylococcus aureus*, *Escherichia coli*, and *Bacillus cereus*, rather than standard reference strains. This work provides a direct proof-of-concept for the application of niosome-encapsulated crocin as a targeted, natural preservative strategy for high-moisture, nutrient-rich foods such as cream pastries, for which effective natural antimicrobial interventions remain limited.

## Materials and methods

### Chemicals and reagents

Crocin (≥ 98% purity) was purchased from Sigma-Aldrich (USA). Span 60 (sorbitan monostearate), Chloroform, methanol, ethanol, and cholesterol (purity, 95%) reagents were obtained from Merck Chemical Co. (Darmstadt, Germany). EDTA, phosphate-buffered saline (PBS), and fetal bovine serum (FBS) were purchased from Gibco (USA). Dialysis membrane (MWCO 12,000 Da) and other chemicals were purchased from Sigma-Aldrich Chemical Co. (St. Louis, MO, USA). All culture media were purchased from Ibresco (Iran), and Reference bacterial strains (*Staphylococcus aureus* ATCC 25923, *Escherichia coli* ATCC 25922, *Bacillus cereus* ATCC 14579) were obtained from the American Type Culture Collection (ATCC). Other analytical solvents and reagents were also sourced from Merck Chemical Co. (Darmstadt, Germany).

### Sample collection

A total of 178 cream pastry samples were randomly collected from local confectioneries in Karaj, Iran, between winter 2023 and summer 2024. All Samples were collected under aseptic and hygienic conditions, labeled with details (purchase location, date, time of sampling, and production time), and immediately transported to the microbiology laboratory at the School of Public Health. Samples were stored at 4 °C until microbiological analysis. Sample collection was conducted to obtain food-derived bacterial isolates rather than to perform a statistical prevalence analysis.

### Bacterial isolation and identification

Cream pastry samples were prepared according to the Iranian National Standard, 2395. For each sample, 10 g was homogenized in 90 mL of sterile Ringer’s solution (1:9 w/v) under aseptic conditions to obtain an initial suspension for microbiological analysis. The homogenized suspensions were serially diluted and inoculated onto selective media: Baird-Parker agar (BPA) for *S. aureus*, *E. coli* broth (EC broth) for *E. coli*, and Mannitol Yolk Polymyxin agar (MYP agar) for *B. cereus*. Plates were incubated at 37 °C for 24–48 h. Suspected colonies were confirmed using biochemical tests: catalase, coagulase, and DNase tests for *S. aureus*; IMViC (indole, methyl red, Voges-Proskauer, citrate) tests for *E. coli*; and lecithinase production and motility tests for *B. cereus*.

The isolates obtained through this protocol were presumptively identified and reported descriptively based on colony morphology, Gram staining, and standardized biochemical profiles in accordance with Iranian National Standard No. 2395. Molecular confirmation methods (e.g., PCR or sequencing) were not aimed to perform in the present study and are acknowledged as a methodological limitation.

### Crocin characterization

Quantitative analysis and purity confirmation of crocin were achieved using High-Performance Liquid Chromatography (HPLC; Agilent 1260 Infinity, USA) equipped with a UV-Vis detector set at 440 nm. Separation was performed on a C_18_ reverse-phase column (250 mm × 4.6 mm, 5 μm particle size). The mobile phase consisted of acetonitrile and water (60:40, v/v) with a flow rate of 1.0 mL/min, and the column temperature was maintained at 30 °C. Injection volume was 20 µL. Crocin samples were dissolved in deionized water at 0.0001% (w/v) concentration and filtered through a 0.22 μm membrane before injection.

### Production and formulation of free and crocin-loaded niosomes

#### Preparation of free crocin solution

A stock solution of crocin (1 mg/mL) was prepared by accurately dispersing 10 mg of pure crocin powder in 10 mL of deionized water. The mixture was stirred at approximately 900 rpm with a magnetic stirrer in the dark for 2 h to ensure complete dissolution and prevent photodegradation. The pH of the solution was maintained at 7.0 throughout the process. Following dissolution, the solution was filtered and centrifuged at 8000 ×g for 15 min to remove any insoluble residues. The resulting clear solution was used for subsequent formulation steps [15].

#### Synthesis of niosome-loaded crocin by thin-hydration method

Crocin-loaded niosomes were synthesized through the thin-film hydration method followed by probe sonication. For a standard 10 mL batch, 700 mg of Span 60 and 150 mg of cholesterol at a 7:3 molar ratio (a composition widely reported to yield stable vesicles for bioactive encapsulation) were accurately weighed and dissolved in a solvent mixture of chloroform and methanol (2:1 v/v; 10 mL chloroform and 15 mL methanol). This mixture was then stirred at 350 rpm for 30 min to ensure uniform blending. The solution was then transferred to a 250 mL round-bottom flask connected to a rotary evaporator (Heidolph Hei-Vap Core, Germany) at 60 °C and 50 rpm under reduced pressure for 20 min, resulting in the formation of a thin lipid film on the inner wall of the flask. Following evaporation, the flask was cooled to room temperature. The lipid film was then hydrated with 10 mL of deionized water containing crocin at a final concentration of 1 mg/mL under continuous rotation at 55 °C and 50 rpm for 1 h without vacuum. The resulting multi-lamellar vesicles (MLV) were subsequently subjected to a size-reduction step using probe sonication (Power Sonic 410, South Korea) at 4 °C for 1 h; a duration established in prior studies to effectively reduce vesicle size without compromising integrity to achieve a uniform, nano-sized dispersion. The dispersion was placed in an ice bath and sonicated using a 6 mm diameter titanium probe with pulsed cycles (30 s ON, 30 s OFF) at 40% amplitude for a total duration of 1 h to obtain a homogeneous suspension of small unilamellar vesicles (SUVs). This established protocol effectively minimizes vesicle size and polydispersity while preventing thermal degradation of crocin and the vesicular structure. For control experiments, empty niosomes (without crocin) were prepared following the identical protocol and molar ratio (7:3 Span 60: Cholesterol), using only the hydration medium (deionized water). The prepared crocin-loaded niosomal dispersions were transferred to sterile vials and stored at 4 °C until further use. This method enables reproducible synthesis of crocin-loaded niosomes with controlled size and properties suitable for food and drug delivery applications [16].

### Characterization of niosome-encapsulated crocin

#### Determination of polydispersity index, zeta potential, and particle size

The particle size and zeta potential of the niosomal formulations were measured at 25 °C using a dynamic light scattering (DLS) (K-ones, scatterscope1, South Korea) system. Particle dispersity was assessed qualitatively based on the shape of the intensity distribution curves. All measurements were performed in triplicate (*n* = 3) and data are presented as mean ± SD. These parameters are essential for assessing the physical stability and homogeneity of niosomal formulations [17].

#### Morphological analysis

Morphology of the crocin-loaded niosomes was examined using Field Emission Scanning Electron Microscopy (FE-SEM) (Mira Tescan, Mira2, Czech Republic). A drop of the niosomal suspension was placed onto a gold-coated aluminum stub and imaged using an FE-SEM at an accelerating voltage of 100 kV. This comprehensive characterization provided detailed insights into the physical properties and structure of the niosomes [18, 19].

#### Fourier transform infrared spectroscopy (FTIR)

To investigate potential molecular interaction between the crocin and niosomal components, Fourier Transform Infrared Spectroscopy (FTIR) (Bruker, Equinox 5, USA) was performed using a Bruker Equinox 5 spectrometer (USA). Lyophilized samples were mixed with potassium bromide (KBr) and compressed into pellets using a hydraulic press. The FTIR analysis was conducted over a spectral range of 4000 to 400 cm ^− 1^ with a constant resolution of 4 cm ^− 1^ at room temperature. The FTIR analysis enabled the identification of functional groups and confirmation of potential chemical interactions [17].

### Determination of standard calibration curve of crocin

To construct the standard calibration curve of crocin, its maximum absorbance wavelength (λmax) was first determined using a UV–Visible spectrophotometer. A stock solution of crocin was prepared at a concentration of 15 mg/mL in deionized water. A mixture of deionized water and methanol was used as the blank to determine the baseline. The absorbance spectrum of the crocin solution was scanned across the wavelength range of 200–900 nm, and λmax was identified at 440 nm, which is consistent with previously reported values for crocin. Subsequently, serial dilutions were prepared from the stock solution using the dilution formula (C₁V₁ = C₂V₂) to obtain working concentrations of 2.14, 2.86, 3.57, 4.29, 5.00, 5.71, and 6.41 mg/mL. The absorbance of each solution was measured at 440 nm, and the values were used to generate the standard calibration curve for crocin [20]. All measurements were performed in triplicate to ensure accuracy and reproducibility .Data were used to generate the standard calibration curve, and linear regression analysis was performed to determine the correlation coefficient (R²) to assess accuracy and reproducibility.

#### Determination of encapsulation efficiency (EE %)

The encapsulation efficiency (EE %) of crocin-loaded niosomes was determined using an ultrafiltration–centrifugation technique. Freshly prepared niosomal formulations were transferred into Amicon Ultra-15 centrifugal filter units (molecular weight cut-off: 12 kDa) and subjected to centrifugation at 10,000 rpm for 60 min at 4 °C using an Eppendorf 580R refrigerated centrifuge to separate the non-entrapped drug from the entrapped drug. This MWCO was selected to ensure retention of niosomes (≈ 139 nm) while allowing free crocin (MW ≈ 976 Da) to pass through. The efficiency of separation was validated in a control experiment where free crocin spiked into an empty niosome suspension was quantitatively recovered in the filtrate. The concentration of free crocin was measured using a UV-Visible spectrophotometer at 278 nm, based on a pre-validated standard calibration curve (R² = 0.999). All measurements were performed in triplicate (*n* = 3) and reported as mean ± SD. The entrapment efficiency (EE %) was calculated using the formula (1).

1 $$\mathrm{EE}\;\%\;=\left[\left(\mathrm A-\mathrm B\right)/\mathrm A\right]\;\times\;100$$

Where A is the total initial crocin concentration in the formulation, and B is the concentration of non-entrapped crocin measured in the filtrate. This method effectively isolated the entrapped crocin, allowing for the precise calculation of entrapment efficiency [21, 22].

#### In vitro release study of crocin from niosomes

In vitro release studies were conducted at pH 1.2 (gastric simulation), 6.8 (representative of dairy-based cream fillings), and 7.2 (physiological conditions) to characterize niosomal release behavior across food-relevant and physiological pH ranges. For this purpose, 1 mL of each crocin-loaded niosome sample was placed in a semipermeable acetate cellulose dialysis bag with a molecular weight cutoff (MWCO) of 12 kDa. The dialysis bag was then immersed in 15 mL of phosphate-buffered saline containing 0.5% (w/v) sodium dodecyl sulfate (PBS–SDS) as the release medium. The dialysis setup was agitated at 100 rpm in a shaker incubator under three pH conditions (1.2, 6.8, and 7.2) at 37 °C for 48 h. At specific time intervals (20 min, 40 min, 1 h, 2 h, 4 h, 8 h, 24 h, and 48 h), 1 mL of the release medium was withdrawn and immediately replaced with an equal volume of fresh pre-warmed PBS-SDS. The concentration of released crocin was determined using a UV–Visible spectrophotometer (BioAquarius, CE7250, UK) at 264 nm. A control experiment with free crocin, having equal concentrations inside and outside the dialysis bag, was performed to account for passive diffusion [23]. Comparisons of cumulative release at different time points and pH values were statistically analyzed using one-way ANOVA followed by Tukey’s post-hoc test, with *p* < 0.05 considered statistically significant.

### Evaluation of antibacterial activity

#### Determination of Minimum Inhibitory Concentration (MIC) and Minimal Bactericidal Concentration (MBC)

MICs for crocin were measured using the broth microdilution method according to Clinical and Laboratory Standards Institute (CLSI) standards guidelines [24]. Bacterial suspensions were adjusted to 0.5 McFarland standard (~ 1.5 × 10⁸ CFU/mL) in sterile Mueller-Hinton broth (MHB) (Merck, Germany). The working inoculum was then further diluted to obtain a final concentration of 5 × 10⁵ CFU/mL in each well, as recommended by CLSI for MIC testing. Then, 100 µL of bacterial suspension was added to each well of a 96-well microplate containing 100 µL of two-fold serial dilution (from 1 to 512 mg/mL) of crocin, resulting in a final volume of 200 µL per well and incubated at 37 ◦C for 18 h. (detailed in Tables 1 and 2). Additionally, controls included a positive control (bacterial suspension and Mueller-Hinton broth) and a negative control (crocin with Mueller-Hinton broth), and empty niosomes (to rule out antimicrobial effects of the carrier itself). Empty niosomes, prepared identically but without crocin, were tested in parallel as a control to account for any potential antimicrobial activity of the vesicle components. The MICs were defined as the lowest concentration of crocin that prevented bacterial growth after 18 h of incubation. All experiments were performed in triplicate (*n* = 3), and results are reported descriptively as stepwise endpoints. MBC was measured by subculturing 10 µL of inoculum from negative wells onto Trypticase Soy Agar (TSA) and then incubating for 24 h at 37 °C. MBC was defined as the lowest concentration of crocin that eliminated bacterial growth, confirmed by the absence of colonies [25].

**Table 1:** Concentrations of free crocin used in MIC/MBC assays (*n* = 3)

Concentration (mg/mL)	Crocin (mg)	Sterile Deionized Water (mL)
1	0.14	199.86
2	0.3	199.7
4	0.5	199.5
8	1.1	198.9
16	2.5	197.5
32	5	195
64	10	190
128	18	182
256	36	164
512	73	127

**Table 2:** Concentrations of niosomal crocin used in MIC/MBC assays (*n* = 3)

Concentration (mg/mL)	Niosomal Crocin (mg)	Sterile Deionized Water (mL)
1	0.05	199.95
2	0.1	199.9
4	0.2	199.8
8	0.5	199.5
16	1	199
32	2	198
64	3.5	196.5
128	7	193
256	14	186
512	28.5	171.5

### Statistical analysis

All quantitative data are presented as mean ± standard deviation (SD) from three independent replicates (*n* = 3). Continuous variables, including particle size, zeta potential, encapsulation efficiency, and crocin release percentages, were analyzed using SPSS software (version 22, IBM, USA). Comparisons between two independent groups (e.g., empty vs. crocin-loaded niosomes) were performed using an unpaired Student’s t-test. Comparisons among more than two groups (e.g., release profiles at different pH values) were conducted using one-way Analysis of Variance (ANOVA) followed by Tukey’s post-hoc test for pairwise comparisons. A p-value < 0.05 was considered statistically significant. MIC and MBC values, being discrete and ordinal, are presented descriptively in tables.

## Results

### Sample collection and microbiological identification

**Fig. 1:**
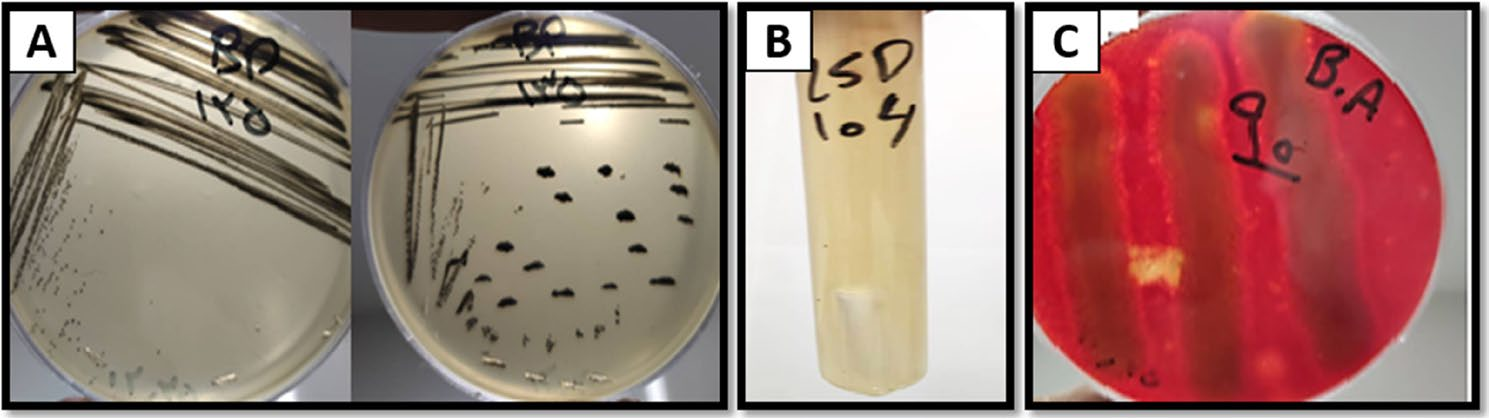
Sample contamination of (**A**) *S.aureus* (**B**) *E.coli* (**C**) *B.cereus*

A total of 178 cream pastry samples were randomly collected from local confectioneries in Karaj, Iran, between winter 2023 and summer 2024. Locations and the number of samples obtained are summarized in Table 3. The sampling strategy was designed to obtain recent, food-derived isolates of major foodborne pathogens rather than to estimate population-level contamination prevalence. Microbiological analyses were conducted to isolate and identify *S. aureus*,* E. coli*, and *B. cereus*. Results indicated that 1.12% of the samples (*n* = 2) were contaminated with coagulase-positive *S. aureus*, while contamination with *E. coli* and *B. cereus* was detected in 0.56% (*n* = 1) of samples (Table 4; Fig. 1A, B, and C). Although, the contamination frequency was low, the isolated strains confirmed the presence of these pathogens in cream pastries and were considered ecologically relevant for subsequent in vitro antibacterial evaluations. These isolates were subsequently used in further experiments, including antibacterial activity assessment. All percentages are based on *n* = 178 total samples, and all isolates were confirmed in triplicate to ensure reproducibility of identification.

**Table 3:** Distribution of the site and number of samples collected

District code	Location (season of sampling)	Number of samples
2	Mohammadshahr (Summer and Winter)	30
2	Meshkindasht (Summer and Winter)	20
12	Mahdasht (Winter)	25
6	Kamalshahr (Summer)	20
4	Fardis Summer and Winter)	35
5	Sarasiab (Winter)	23
5	Marlik (Summer)	10
9	Valadabad (Winter)	10
9	Zibadasht (Winter)	5

**Table 4:** Results of microbiological analysis of cream pastry sample

Sample type	Sample code	The standard Contamination rate %	Type of contamination
Cream pastry	125	1.12	*S.aureus*
Cream pastry	135	1.12	*S.aureus*
Cream pastry	106	0.56	*E.coli*
Cream pastry	90	0.56	*B.cereus*

### Characterization of crocin

**Fig. 2:**
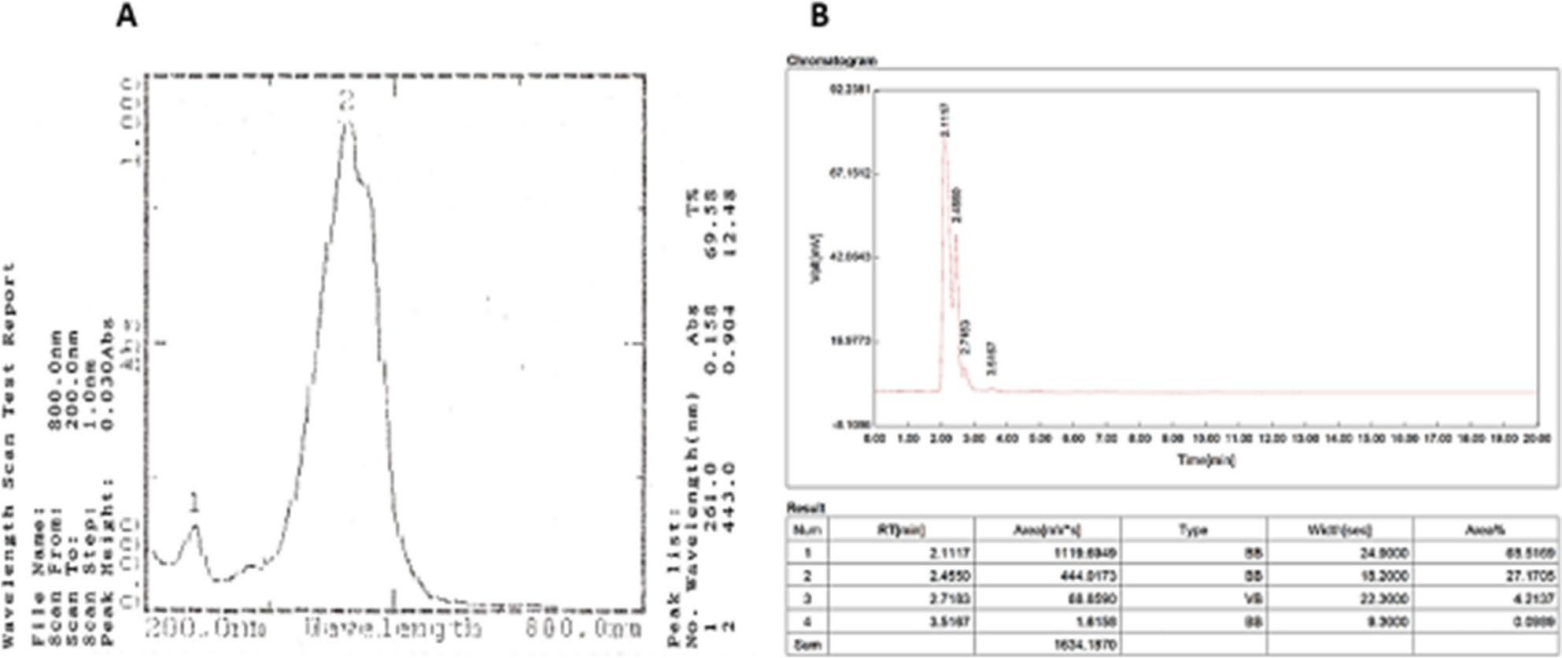
**A** UV/Visible spectra of 0.0001% (1 mg/L) of crocin (200–800 nm) (**B**) HPLC analysis of crocin at 440 nm (concentration: 0.0001% or 1 mg/L)

Crocin was characterized using UV-Visible spectroscopy and HPLC to confirm its structural integrity, purity, and concentration. Ultraviolet–Visible (UV–Vis) spectra were recorded with a Shimadzu UV-1800 spectrophotometer over the range 200–800 nm, showing a characteristic absorption peak at 440 nm for a 0.0001% (1 mg/L) aqueous crocin solution (Fig. 2A). All UV–Vis measurements were performed in triplicate (*n* = 3), and results are presented as mean ± SD to ensure reproducibility. The HPLC chromatogram displayed three main peaks corresponding to crocin-1 (α-crocin), crocin-2, and crocin-3. These peaks were consistently observed in replicate samples, confirming the presence and purity of multiple crocin isomers in the solution (Fig. 2B).

### Characterization of niosomes-loaded crocin

Dynamic Light Scattering (DLS) analysis was performed to determine the particle size distribution and homogeneity of the niosomal formulations. The DLS intensity distribution curves revealed a narrow and unimodal size distribution for crocin-loaded niosomes, indicating low polydispersity and good uniformity of the vesicular system. This homogeneous particle size distribution suggests successful formulation and physical stability of the niosomal carrier, which is essential for reproducible antimicrobial performance.

**Fig. 3:**
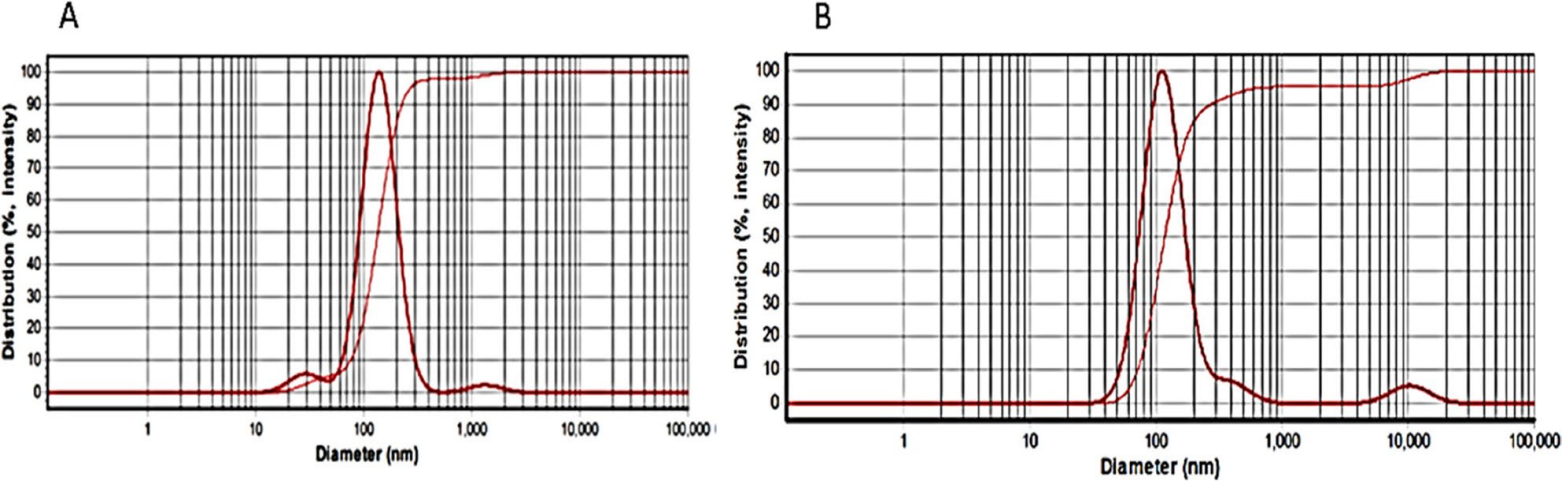
DLS graph of (**A**) crocin-loaded niosome, (**B**) crocin-free niosome

**Fig. 4:**
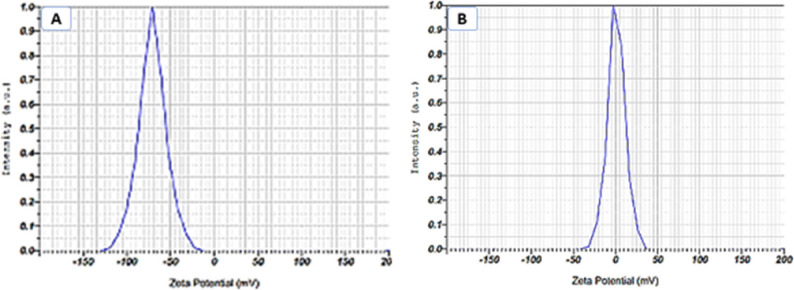
Zeta potential of (**A**) crocin-free niosome, (**B**) crocin-loaded niosome

The mean hydrodynamic diameter of crocin-loaded niosomes was determined to be 139 ± 0.7 nm (Fig. 3A), whereas crocin-free niosomes displayed a smaller average size of 113 ± 0.42 nm (Fig. 3B). All measurements were performed in triplicate (*n* = 3) to ensure reproducibility. The observed increase in particle size following crocin loading is consistent with the incorporation of crocin within the niosomal bilayer. This interpretation is further supported by the corresponding shift in zeta potential values (Fig. 4) and the high encapsulation efficiency (72 ± 0.5%) obtained experimentally [26].

#### Zeta potential

The zeta potential of crocin-loaded niosomes was determined to be + 9.9 ± 0.5 mV (Fig. 4A), in contrast to -70.9 ± 0.8 mV observed for crocin-free niosomes (Fig. 4B). Typically, a higher absolute value of zeta potential, whether positive or negative, is indicative of enhanced colloidal stability [27]. The strongly negative zeta potential of the crocin-free niosomes, which is at the more negative end of the range commonly reported for Span 60/cholesterol-based vesicular systems, may be associated not only with the presence of hydroxyl (− OH) groups in Span 60 but also with the intensive probe sonication applied during vesicle size reduction. High-energy sonication has been reported to promote surface reorganization and increased exposure of polar surfactant head groups, thereby amplifying the apparent surface charge. The notable shift of the zeta potential toward a near-neutral/slightly positive value following crocin encapsulation suggests a substantial modification of the vesicle interfacial properties rather than a measurement artifact [13, 18]. Given the highly polar and glycosylated structure of crocin, its incorporation into the niosomal bilayer may partially screen or neutralize native negative charges and alter the effective shear plane sensed during electrophoretic mobility measurements [28]. This shift, therefore, reflects interactions between crocin and the niosomal membrane, likely through interfacial association or partial embedding of crocin molecules, leading to modified surface charge characteristics and potentially influencing the colloidal stability and biological behavior of the nanoparticles, as reported for other bioactive-loaded vesicular systems [18, 26].

#### FE-SEM microscopy

**Fig. 5:**
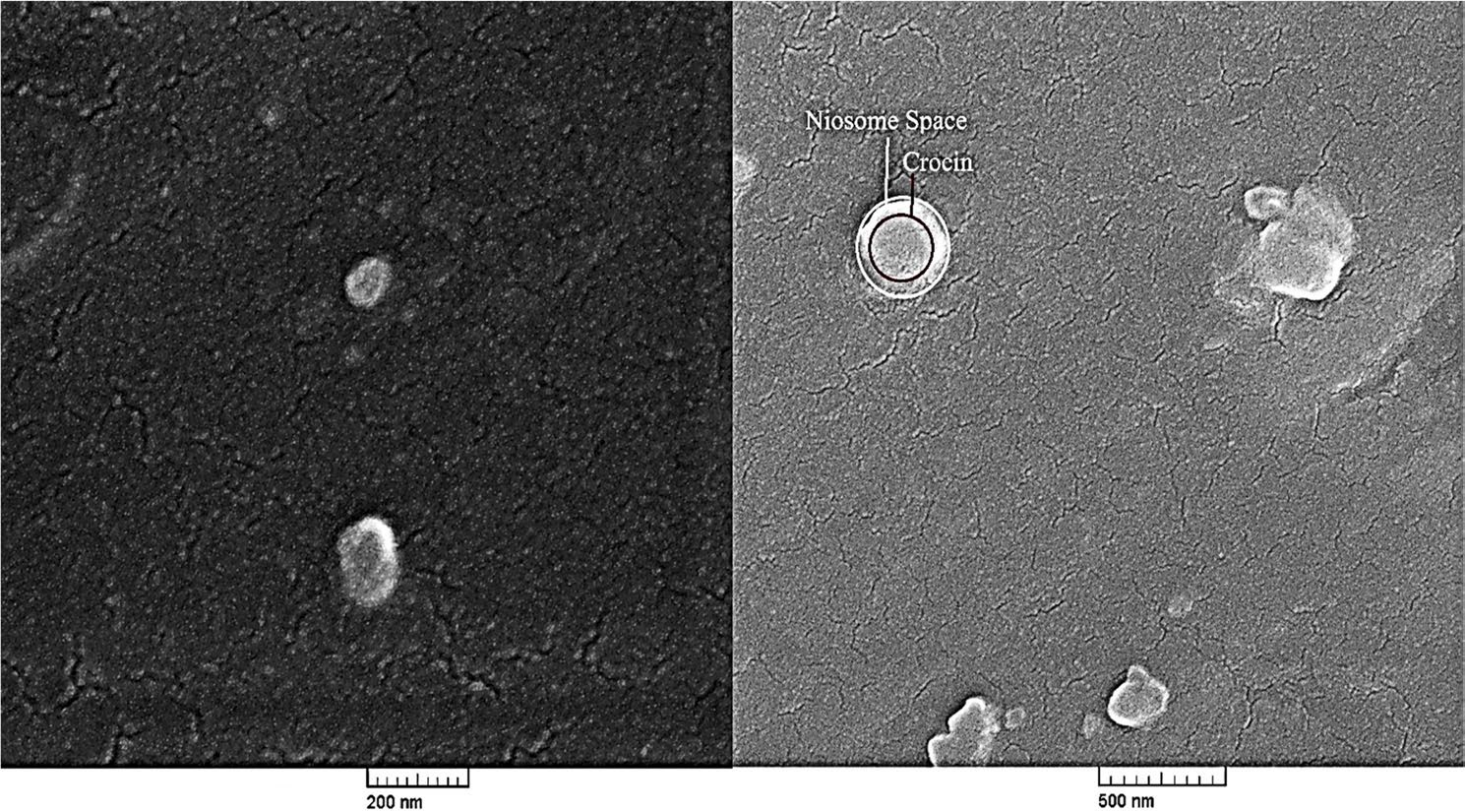
FE-SEM image of crocin-loaded niosome

Field Emission Scanning Electron Microscopy (FE-SEM) was utilized to carefully analyze the surface morphology and structural features of the crocin-loaded niosomes. For sample preparation, a small aliquot of the niosomal suspension was evenly dropped onto a clean aluminum foil substrate and allowed to air dry in ambient conditions. To enhance the conductivity and prevent charging effects during imaging, the dried samples were sputter-coated with a thin layer (~ 5 nm) of gold. Figure 5 presents an SEM image of the crocin-loaded niosomal formulation at two different magnifications with scale bars of 200 nm and 500 nm. The images reveal that the niosomes predominantly exhibit spherical morphology with smooth, intact surfaces, indicating structural integrity. The vesicles are well-dispersed with no significant aggregation, which reflects the successful encapsulation and structural stability of the niosomal system. A qualitative assessment based on the scale bar indicates particle sizes within an approximate range of 100–200 nm, which is consistent with the quantitative mean hydrodynamic diameter of 139 ± 0.7 nm measured by DLS (Fig. 3A). SEM thus provides visual confirmation of morphology and dispersion, while DLS delivers statistically robust, quantitative size data. The observed uniformity in shape and size distribution supports the effectiveness of the thin-film hydration combined with sonication techniques used in the preparation process. These physical characteristics corroborate the successful fabrication of nano-sized, uniform, and stable crocin-loaded niosomes [28].

#### Fourier-Transform Infrared (FTIR) spectroscopy

**Fig. 6:**
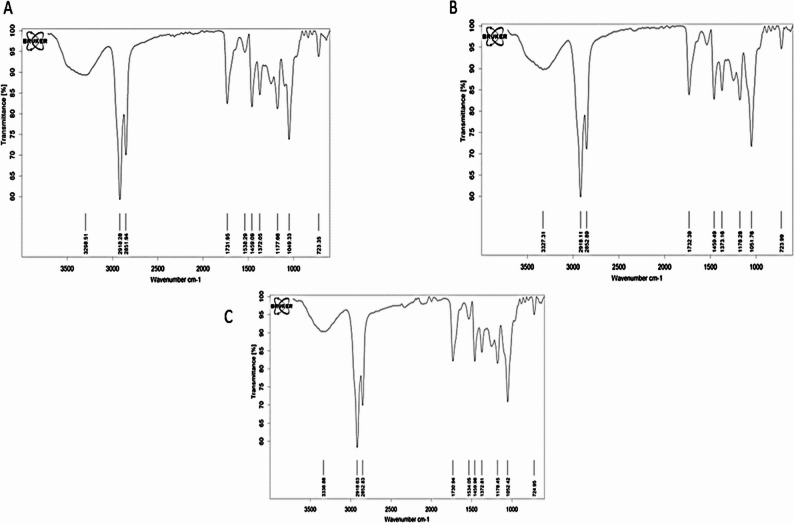
FTIR spectrum (**A**) crocin, (**B**) crocin-loaded niosome, (**C**) empty niosome

FTIR spectroscopy was employed to characterize and compare the molecular structures of free crocin, crocin-loaded niosomes, and free niosomes. The resulting spectra revealed strong similarities, indicative of shared structural components and common functional groups across these formulations (Fig. 6). All spectra displayed a relatively flat baseline, reflecting high-quality data acquisition. A prominent broad absorption band was consistently observed around 3298 to 3337 cm⁻¹ (specifically 3298.51 cm⁻¹ for crocin (Fig. 6A), 3327.31 cm⁻¹ for crocin-loaded niosomes (Fig. 6B), and 3336.88 cm⁻¹ for free niosomes (Fig. 6C), characteristic of extensive hydrogen bonding attributed to hydroxyl (O–H) and/or amine (N–H) groups [29]. Sharp peaks near 2918 cm⁻¹ and 2852 cm⁻¹, observed uniformly across all samples, correspond to C–H stretching vibrations of aliphatic methylene (CH₂) and methyl (CH₃) groups.

Each spectrum also exhibited a medium-intensity peak between 1731 and 1732 cm⁻¹, assigned to carbonyl (C = O) stretching, consistent with previously reported FTIR spectra of crocin [29]. The fingerprint region (1550–700 cm⁻¹) presented a complex pattern of absorption peaks at approximately 1530–1534, 1459–1460, 1372–1373, 1177–1178, and 1049–1052 cm⁻¹, reflecting diverse bending and stretching vibrations. Additionally, a peak around 723–725 cm⁻¹ confirmed the presence of aromatic moieties [30, 31].

Notably, subtle variations in peak positions and intensities within the fingerprint region, along with minor shifts in the broad O–H/N–H stretching bands, distinguished the three spectra. These differences likely arise from variations in functional group orientation or specific substituents unique to each molecular environment.

In summary, FTIR analysis indicates that the characteristic functional groups of both crocin and the niosomal components (Span 60, cholesterol) are present in the loaded formulation, with no evidence of destructive chemical bonding. The observed subtle spectral variations, particularly in the fingerprint region, are suggestive of physical interactions (e.g., hydrogen bonding, van der Waals forces) between crocin and the niosomal bilayer, which is consistent with successful physical encapsulation and stabilization of the compound within the vesicles [28]. Thus, while FTIR provides supportive evidence of physical incorporation without chemical alteration, the direct proof of successful encapsulation remains the quantitative encapsulation efficiency measurement, the significant shift in zeta potential, and the increase in hydrodynamic diameter.

### Standard calibration curve of crocin

**Fig. 7:**
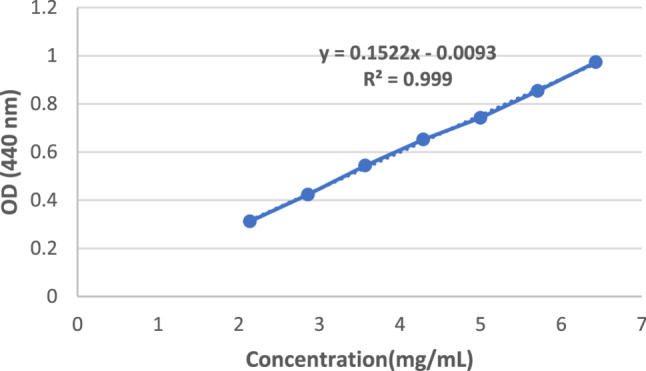
Standard calibration curve of the crocin

A calibration curve for crocin quantification was constructed by measuring the absorbance (A) at 440 nm across a concentration range of 2.14 to 6.43 mg/mL (Fig. 7). The measured absorbance values increased linearly with crocin concentration, indicating a strong positive linear correlation between crocin concentration and absorbance. The linear regression equation derived from the data is (2).

2 $$\mathrm Y=0\cdot1516\mathrm X+0\cdot0516\;\left(\mathrm R2\;=\;0\cdot999\right)$$

Where Y represents the absorbance and X is the concentration of crocin in mg/mL.

The high coefficient of determination (R 2 = 0.999) indicates linearity, the high sensitivity, reproducibility, and reliability of the method over the tested concentration range. The calibration curve (Fig. 7) provides a robust quantitative basis for evaluating encapsulation efficiency and release of crocin in both free form and when encapsulated within nano-niosomes.

#### Encapsulation Efficiency (EE %)

Spectrophotometric determination of the entrapment efficiency (EE) of crocin-loaded niosomes was made using a standard calibration curve; absorbance (Y) was linearly correlated with crocin concentration (X), based on Eq. (3).

3 $$\mathrm Y=0.1516\mathrm X+0.0516\;\left(\mathrm R^2\;=\;0\cdot999\right)$$

After centrifugation of the niosomal dispersion, the absorbance of the supernatant at crocin’s maximum absorption wavelength (278 nm) was measured by UV-Vis spectrophotometry, and the concentration of unencapsulated crocin was calculated by substituting the absorbance measurement in the standard curve Eq. (22).

The entrapment efficiency was estimated as a percentage of entrapped crocin (wₑ) to the total initial crocin (wₜ) and was based on the following Eq. (4).

4 $$\mathrm{EE}\left(\%\right)\;=\left({\mathrm w}_{\mathrm e}/\;{\mathrm w}_{\mathrm t}\right)\;\times\;100$$

Results indicated that crocin-loaded niosomal formulation provided an entrapment efficiency of 72 ± 0.5% of crocin, suggesting that crocin was effectively incorporated into niosomal vesicles. This relatively high EE value may be attributed to the amphiphilic nature of crocin and its favorable interaction with the bilayer components of the niosomes [21].

#### Crocin release from niosomes

**Fig. 8:**
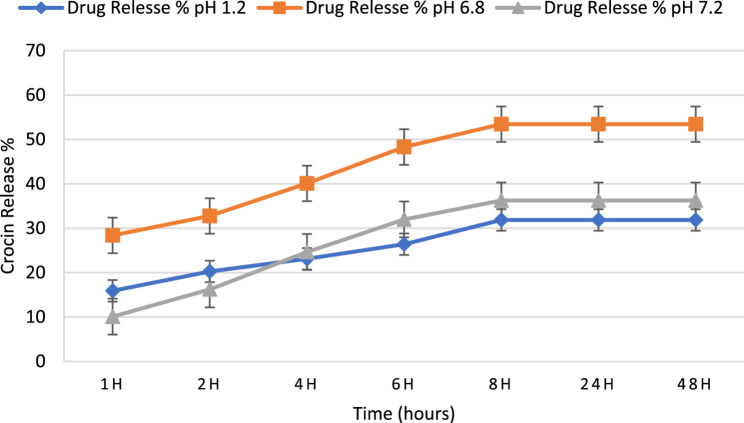
Release profile of niosome-encapsulated crocin in PBS medium (pH 1.2, 6.8, 7.2)

The release of crocin from niosomal formulations was evaluated over a period of 48 h at three different pH conditions: 1.2 (strongly acidic), 6.8 (mildly acidic), and 7.2 (neutral). As shown in Fig. 8, crocin release exhibited a sustained and controlled pattern in all conditions. At pH 6.8, crocin displayed the highest release rate, with 28.38% released at 1 h, increasing steadily to 53.44% by 8 h, and plateauing thereafter to 48 h. This enhanced release under mildly acidic conditions may be attributed to increased bilayer fluidity and partial loosening of the nonionic surfactant packing, which enhances membrane permeability and facilitates crocin diffusion from the niosomal vesicles.

At pH 1.2, the release was slower, starting at 15.90% at 1 h and reaching 31.86% at 8 h, maintaining this level up to 48 h. Under strongly acidic conditions, protonation of the polar head groups of the nonionic surfactants leads to a more compact and ordered bilayer structure, accompanied by reduced membrane hydration and fluidity. This structural tightening significantly limits membrane permeability and hinders the diffusion of crocin from the niosomal vesicles, resulting in a restrained release profile [23, 37]. The slowest release was observed at pH 7.2, with 10.09% released at 1 h and 36.24% at 8 h, stabilizing after that. At neutral pH, the niosomal bilayer attains a more stable and energetically favorable organization, characterized by stronger hydrophobic interactions between crocin molecules and the surfactant bilayer. This enhanced stability promotes higher drug retention within the vesicles and consequently slows the release rate compared to acidic conditions [18, 23]. These data indicate a pH-dependent release, with maximal release under mildly acidic conditions and reduced release under strongly acidic and neutral environments. The sustained release profile across all pH values confirms the potential of niosomal carriers to provide controlled drug release over extended timescales [23].

### MIC and MBC determination

Crocin’s antibacterial activity was evaluated against *S. aureus*, *E. coli*, and *B. cereus* using the microdilution method. The results, reported in Table 5, showed that crocin, both in its free form and encapsulated in niosomes, had an inhibitory effect on the studied bacteria. Niosomal crocin exhibited lower MIC and MBC values against *S. aureus* and *E. coli* compared to free crocin, showing a two-fold reduction in MIC. In contrast, no difference in MIC or MBC values was observed for *B. cereus*. These results indicate an improved antibacterial efficacy of crocin upon niosomal encapsulation against selected bacterial strains. Moreover, the lower MIC values ​​for crocin encapsulated in niosomes compared to the free form showed that this encapsulated form of crocin can be more effective against the tested strains [32]. Importantly, control experiments with empty niosomes showed no inhibitory activity against any of the tested strains, confirming that the observed effects were due to crocin alone.

**Table 5:** MIC and MBC of free and niosomal crocin against selected bacterial strains

Bacterial strain	MIC of free crocin (mg/mL)	MIC of crocin encapsulated in niosomes (mg/mL)	MBC of free crocin (mg/mL)	MBC of crocin encapsulated in niosomes (mg/mL)
*S. aureus*	128	64	256	128
*E. coli*	128	64	256	128
*B. cereus*	256	256	512	512

MIC and MBC values were determined using the broth microdilution method and are reported as discrete concentration endpoints. Values represent comparative measurements from independent experiments. Empty niosomes showed no inhibitory activity against the tested bacterial strains

## Discussion

Foodborne diseases remain a major global public health concern, posing significant risks to consumers [33]. Although, chemical preservatives have long been used to inhibit microbial growth and extend shelf life [4], growing awareness of the impact of dietary components on human health has raised demand for safer, more natural alternatives [3]. Indeed, several synthetic preservatives are associated with adverse health effects; for example, sulfites have been reported to degrade vitamin B₁ (thiamine) in food products [5]. Consequently, bioactive compounds from natural sources have attracted considerable interest as potential antimicrobial agents for food preservation, offering the dual benefits of consumer safety and eco-friendliness [8]. The present study addressed an overlooked yet critical area: microbial contamination in cream pastries, a product highly susceptible to spoilage due to its moisture content and nutrient-rich composition. Among the 178 samples collected from confectioneries in southern Karaj, contamination with *S. aureus*,* E. coli*, and *B. cereus* was confirmed, underscoring the need for effective antimicrobial interventions in such products. It should be noted that the microbiological survey was not intended as a prevalence or epidemiological assessment; rather, it was conducted to obtain ecologically relevant, food-derived isolates for evaluating the antibacterial performance of the proposed niosomal formulation. Therefore, contamination frequencies are reported descriptively without inferential statistical comparison. While the contamination rate was low, even sporadic occurrences of these pathogens in ready-to-eat, high-moisture products such as cream pastries represent a potential public health concern due to their widespread consumption. Our results demonstrated that crocin exhibited measurable antibacterial activity against these isolates, consistent with previous reports of saffron-derived compounds inhibiting a wide range of foodborne pathogens [8, 34]. Saffron (*Crocus sativus* L.) is valued worldwide not only for its culinary properties but also for its pharmacological activities [9]. The petals, often considered a byproduct, contain bioactive constituents with antioxidant and antimicrobial potential [10]. Crocin, the major water-soluble carotenoid in saffron, is particularly noteworthy for its broad-spectrum antibacterial activity, which includes efficacy against Gram-negative bacteria, an effect often attributed in part to the presence of its hydroxyl (-OH) groups [9]. However, its direct application in food systems can be limited by environmental instability and interactions with other food components.

Encapsulation technologies, such as niosomes, have emerged as highly promising carriers for bioactive compounds, including phenolics, terpenoids, and carotenoids. Niosomes are non-ionic surfactant-based bilayer vesicles that encapsulate active agents, conferring protection from environmental degradation, improved solubility, and controlled release profiles, thereby enhancing bioavailability and efficacy [12]. Crucially, the nanoscale dimensions of niosomal vesicles (~ 100–200 nm) facilitate intimate interactions with microbial surfaces and promote penetration across bacterial cell envelopes. This nanosize advantage increases the local concentration of the bioactive compound at targeted sites, potentially disrupting cellular membranes and biofilm matrices more effectively than free compounds [18]. Recent studies have validated the improved antimicrobial profile of niosome-encapsulated agents, such as Glycyrrhiza glabra extract [26] and rosmarinic acid [23], as well as antibiotics like ciprofloxacin and vancomycin, underscoring the broad-spectrum applicability of this delivery platform [35, 36].

In the present study, crocin encapsulation with a high encapsulation efficiency (72 ± 0.5%) led to a clear shift in surface charge compared with empty niosomes (-70.9 ± 0.8 mV), resulting in a moderately positive zeta potential of crocin-loaded niosomes (+ 9.9 ± 0.5 mV). This change, together with the relatively small and uniform particle size (139 ± 0.7 nm), suggests the formation of a physically stable nanosystem and confirms the successful incorporation of crocin into the niosomal structure. This change, together with the relatively small and uniform particle size (139 ± 0.7 nm), suggests the formation of a physically stable nanosystem and confirms the successful incorporation of crocin into the niosomal structure. These properties synergistically enabled sustained crocin release and enhanced antibacterial activity against *S. aureus* and *E. coli* isolates (approximately two-fold lower MIC/MBC values compared with free crocin), consistent with prior reports on saffron-derived antimicrobials [8, 34]. A comprehensive interpretation of the MIC and MBC results provides deeper insight into the antibacterial behavior of crocin in both free and niosome-encapsulated forms. The minimum inhibitory concentration (MIC) represents the lowest concentration that prevents visible bacterial growth, while the minimum bactericidal concentration (MBC) is defined as the lowest concentration that kills ≥ 99.9% of the bacterial inoculum, indicating bactericidal action rather than just growth inhibition. MBC/MIC ratios ≤ 4 are generally interpreted as indicative of bactericidal activity, whereas larger ratios suggest a primarily bacteriostatic effect [24]. 

For *Staphylococcus aureus* and *Escherichia coli* treated with crocin-loaded niosomes, the MBC values were approximately two-fold higher than the corresponding MIC values, indicating that higher concentrations were required to achieve bacterial killing after growth inhibition. This pattern is consistent with vesicular delivery systems, where sustained release prolongs exposure and may facilitate a transition from bacteriostatic to bactericidal effects at elevated concentrations. Such a phenomenon has been reported for other nano-encapsulated antimicrobial agents and is attributed to improved penetration and retention at the bacterial surface despite intrinsic physicochemical barriers. (14،18) In the case of *Bacillus cereus*, identical MIC and MBC values were observed for both free and niosome-encapsulated crocin. Although *B. cereus* is capable of spore formation, the MIC/MBC tests in this study were conducted on actively dividing cells, whose cytoplasmic membrane and wall integrity may be directly compromised by crocin. Moreover, phenotypic characteristics of certain Gram-positive bacteria can make them more susceptible to direct antibacterial killing, resulting in overlapping MIC and MBC values, as has been documented for other natural antimicrobial agents [2, 8].

Furthermore, the observation that MIC and MBC values for *E. coli* and *S. aureus* were comparable between free and encapsulated crocin suggests that niosomal encapsulation did not fundamentally alter the intrinsic antibacterial potency of crocin under the test conditions. Instead, encapsulation improved colloidal stability, dispersion uniformity, and controlled release, enhancing functional efficacy without artificially lowering MIC/MBC values. Similar trends have been reported for other nanoencapsulated phytochemicals (15،13). Notably, encapsulating crocin within niosomes substantially enhanced its inhibitory effects against *S. aureus* and *E. coli* compared to its free form. This improvement may be attributed to several factors: (i) protection of crocin from environmental degradation, (ii) sustained release at the site of action, and (iii) enhanced penetration of the bacterial cell wall due to the nanoscale size of the vesicles. The nano-vesicular structure increases crocin’s effective contact with bacterial membranes and may facilitate penetration into biofilms, contributing to the improved microbicidal effects observed [36].

pH-dependent release studies revealed that crocin-loaded niosomes exhibited quantitatively distinct release profiles across different pH values, which supports the adaptability of these nanocarriers to varying food environments. Interestingly, crocin-loaded niosomes released slightly more in neutral conditions (36.2%) than in acidic conditions (31.8%), which differs from some reports, such as Ghanbarzadeh et al. [37], who found higher release rates in acidic environments. This variation may reflect differences in vesicle composition, drug–surfactant interactions, or the hydrophilic nature of crocin [37]. While our in vitro release studies under standardized pH conditions provide essential proof-of-concept for the pH-responsive behavior and stability of crocin-loaded niosomes, the true efficacy of this strategy for food preservation must be validated in complex food matrices. Factors such as fat content, protein interactions, and water activity in real cream pastries could influence niosome stability, crocin release kinetics, and antimicrobial activity. Therefore, future applied research should focus on evaluating the performance of this nano-encapsulated system within relevant food simulants or directly in inoculated food products under realistic storage conditions.

The enhanced antimicrobial activity observed with crocin-loaded niosomes aligns with the growing body of evidence supporting nanocarrier systems as effective tools for boosting the potency of natural antimicrobials. Importantly, given the increasing prevalence of antibiotic-resistant pathogens, natural compounds such as crocin—especially when delivered via advanced encapsulation systems—offer a sustainable dual strategy: it preserves food safety while mitigating the risks associated with synthetic preservatives and conventional antimicrobials. Such nanocarrier systems strike a balance between efficacy, safety, and consumer acceptance, thereby addressing critical gaps in current food preservation technologies [38]. Despite the promising findings, this study has certain limitations that should be acknowledged. Antibacterial efficacy and release kinetics were assessed under controlled in vitro conditions, and no inferential comparisons were performed across food matrices. Additionally, bacterial identification was based on phenotypic and biochemical characterization, and molecular confirmation methods were not included in this study, which should be addressed in future work to further strengthen species-level identification. Finally, the long-term stability of the niosomes and the potential influence of food processing or storage conditions on their integrity and activity require further assessment before practical application can be considered.

Overall, this study confirms the antimicrobial potential of crocin and demonstrates that niosomal encapsulation enhances its stability and efficacy. Importantly, crocin-loaded niosomes were effective against bacterial contaminants from cream pastries, highlighting their potential as a natural, nanotechnology-based alternative to conventional preservatives.

## Conclusions

This study demonstrated the modest inherent antibacterial activity of crocin against bacterial strains isolated from cream pastry samples under in vitro conditions. Encapsulation of crocin within niosomes resulted in approximately two-fold reductions in MIC/MBC values against *S.aureus* and *E.coli* compared with free crocin (MIC range: 128–256 mg/mL), highlighting the proof-of-concept potential of nanocarrier systems to enhance antibacterial performance and stability. These results provide foundational in vitro evidence for the antibacterial potential of crocin-loaded niosomes, while their efficacy in complex food matrices and the mechanisms underlying their activity, including biofilm disruption and membrane interactions, remain to be determined. Although crocin demonstrates variable efficacy depending on purity, source, and experimental conditions, encapsulation within niosomes offers a promising strategy to enhance stability and antimicrobial performance. Given the rising global challenge of multidrug-resistant bacteria, these findings highlight the potential of nano-encapsulated crocin as a natural antimicrobial agent, emphasizing the need for comprehensive studies to evaluate its safety, functional stability, and practical applicability in real food systems.

## Supplementary Information

Supplementary Material 1.

Supplementary Material 2.

Supplementary Material 3.

## Data Availability

The datasets used and/or analyzed during the current study are available from the corresponding author on reasonable request.

## Abbreviations

*B. cereus*: *: Bacillus cereus*
DLS: Dynamic Light Scattering
*E. coli*: *Escherichia coli*
*EC* broth: *E. coli* broth
EE: Encapsulation Efficiency
FBS: Fetal Bovine Serum
FE-SEM: Field Emission Scanning Electron Microscopy
FTIR: Fourier Transform Infrared Spectroscopy
HPLC: High-Performance Liquid Chromatography
IMViC: Indole, Methyl Red, Voges-Proskauer, Citrate
MBC: Minimum Bactericidal Concentration
MHB: Mueller-Hinton Broth
MIC: Minimum Inhibitory Concentration
MYP: Mannitol Yolk Polymyxin
MWCO: Molecular Weight Cut-Off
PBS: Phosphate-Buffered Saline
PDI: Polydispersity Index
*S. aureus*: *Staphylococcus aureus*
SDS: Sodium Dodecyl Sulfate
SEM: Scanning Electron Microscopy
TSA: Trypticase Soy Agar
UV-Vis: Ultraviolet-Visible Spectroscopy
