# Supplementary material for: Antibacterial activity of crocin-loaded niosomes against foodborne pathogens isolated from cream pastries
Source: BMC Microbiol. 2026 Apr 25;26:531. doi: 10.1186/s12866-026-05059-8 (PMC13238057; doi:10.1186/s12866-026-05059-8)
Supplement: Supplementary file 1 — Supplementary Material 1. [file 12866_2026_5059_MOESM1_ESM.docx]

**
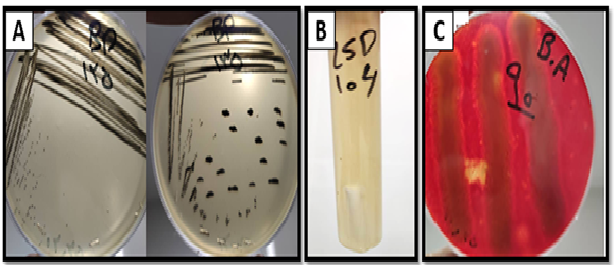
**

**Fig1.** Sample contamination of **A)** *S.aureus* **B)** *E.coli* **C)** *B.cereus*


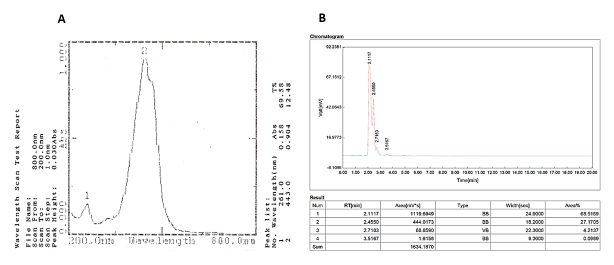


**Fig 2.** **(a)** UV/Visible spectra of 0.0001 % (1 mg/L) of crocin (200-800 nm) (**b)** HPLC analysis of crocin at 443 nm (concentration: 0.0001 % or 1 mg/L)


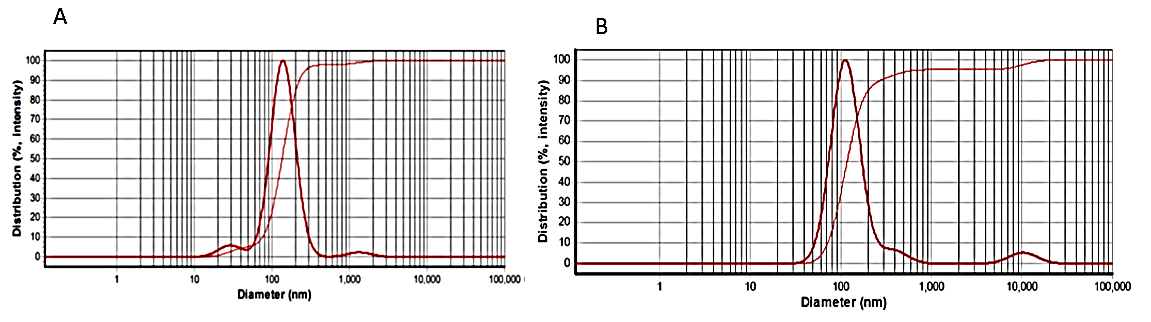


**Fig 3.** DLS graph of **A)** crocin-loaded niosome, **B)** crocin-free noisome

**
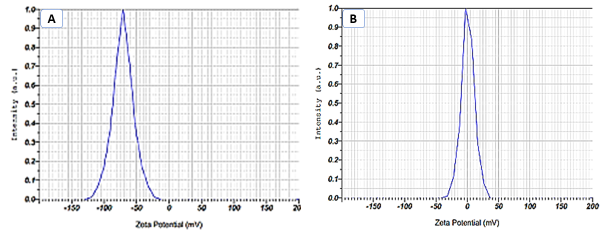
**

**Fig 4.** zeta potential of **A)** crocin-free niosome, **B)** crocin-loaded niosome

**
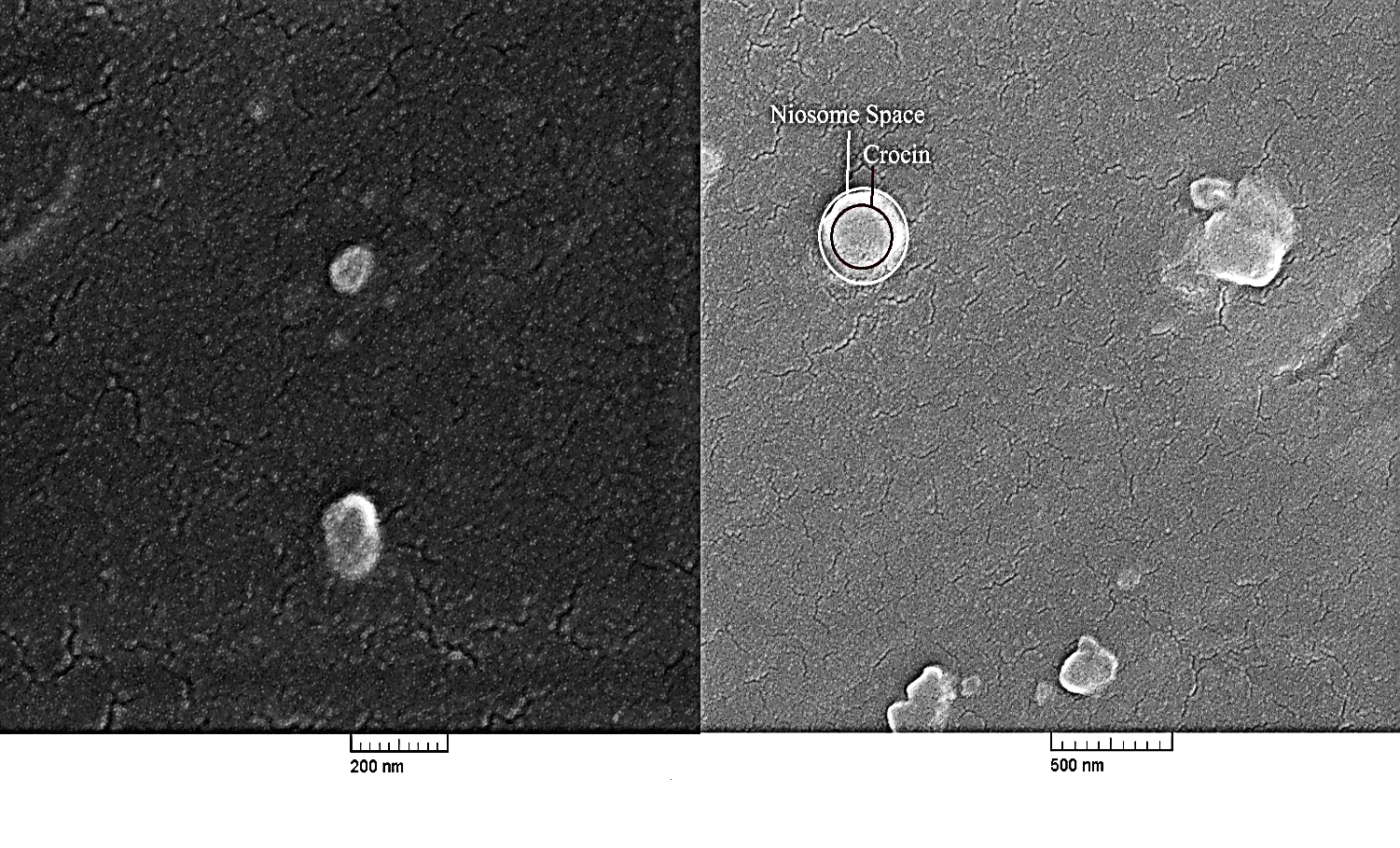
**

**Fig 5.** FE-SEM image of crocin-loaded noisome


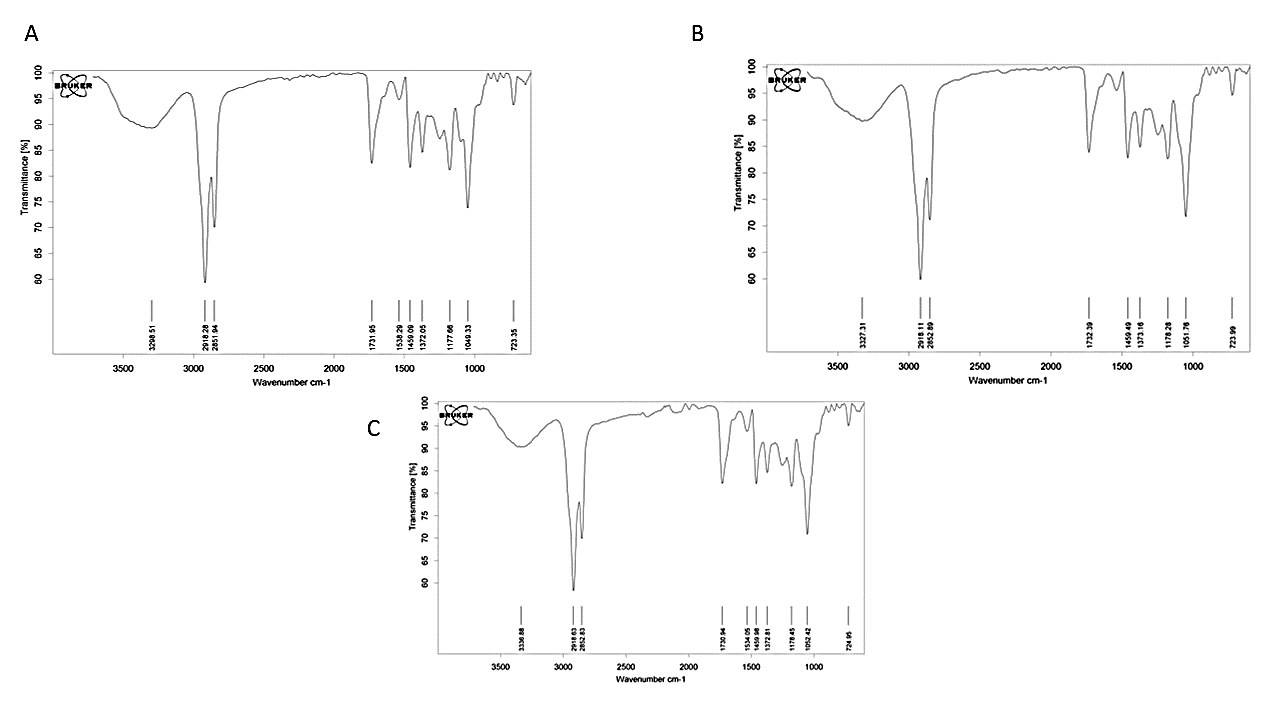


**Fig 6.** FTIR spectrum **A)** crocin, **B)** crocin-loaded niosome, **C)** free crocin noisome

**Fig 7.** Standard calibration curve of the crocin

**Fig 8.** Release profile of niosome-encapsulated crocin in PBS medium (pH **1.2, 6.8, 7.2**)
