# Supplementary material for: Antibacterial activity of crocin-loaded niosomes against foodborne pathogens isolated from cream pastries
Source: BMC Microbiol. 2026 Apr 25;26:531. doi: 10.1186/s12866-026-05059-8 (PMC13238057; doi:10.1186/s12866-026-05059-8)
Supplement: Supplementary file 2 — Supplementary Material 2. [file 12866_2026_5059_MOESM2_ESM.docx]

**Table 1**. Concentrations of free crocin

| **Concentration(mg/mL)** | **Crocin (mg)** | **Sterile Deionized Water (mL)** |
| --- | --- | --- |
| 1 | 0.14 | 199.86 |
| 2 | 0.3 | 199.7 |
| 4 | 0.5 | 199.5 |
| 8 | 1.1 | 198.9 |
| 16 | 2.5 | 197.5 |
| 32 | 5 | 195 |
| 64 | 10 | 190 |
| 128 | 18 | 182 |
| 256 | 36 | 164 |
| 512 | 73 | 127 |

| **Concentration(mg/mL)** | **Niosomal Crocin (mg)** | **Sterile Deionized Water (mL)** |
| --- | --- | --- |
| 1 | 0.05 | 199.95 |
| 2 | 0.1 | 199.9 |
| 4 | 0.2 | 199.8 |
| 8 | 0.5 | 199.5 |
| 16 | 1 | 199 |
| 32 | 2 | 198 |
| 64 | 3.5 | 196.5 |
| 128 | 7 | 193 |
| 256 | 14 | 186 |
| 512 | 28.5 | 171.5 |

**Table 2.** Concentrations of niosomal crocin

**Table 3.** Distribution of the site and number of samples collected

| **District code** | **Location (season of sampling)** | **Number of samples** |
| --- | --- | --- |
| 2 | Mohammadshahr (Summer and Winter) | 30 |
| 2 | Meshkindasht (Summer and Winter) | 20 |
| 12 | Mahdasht (Winter) | 25 |
| 6 | Kamalshahr (Summer) | 20 |
| 4 | Fardis Summer and Winter) | 35 |
| 5 | Sarasiab (Winter) | 23 |
| 5 | Marlik (Summer) | 10 |
| 9 | Valadabad (Winter) | 10 |
| 9 | Zibadasht (Winter) | 5 |

**Table 4.** Results of microbiological analysis of cream pastry sample

| **Sample type** | **Sample code** | **Contamination rate %0** | **Type of contamination** |
| --- | --- | --- | --- |
| Cream pastry | 125 | 1.12 | *S.aureus* |
| Cream pastry | 135 | 1.12 | *S.aureus* |
| Cream pastry | 106 | 0.56 | *E.coli* |
| Cream pastry | 90 | 0.56 | *B.cereus* |

**Table 5.** MIC and MBC of crocin in free form and encapsulated in niosomes against the tested bacteria. Values are presented as means ± standard deviation.

| **Bacterial strain** | **MIC of free crocin (mg/ml)** | **MIC of crocin encapsulated in niosomes (mg/ml)** | **MBC of free crocin (mg/ml)** | **MBC of crocin encapsulated in niosomes (mg/ml)** |
| --- | --- | --- | --- | --- |
| ***S. aureus*** | 128 | 64 | 256 | 128 |
| ***E. coli*** | 128 | 64 | 256 | 128 |
| ***B. cereus*** | 256 | 256 | 512 | 512 |
