# Supplementary material for: Antibacterial activity of crocin-loaded niosomes against foodborne pathogens isolated from cream pastries
Source: BMC Microbiol. 2026 Apr 25;26:531. doi: 10.1186/s12866-026-05059-8 (PMC13238057; doi:10.1186/s12866-026-05059-8)
Supplement: Supplementary file 3 — Supplementary Material 3. [file 12866_2026_5059_MOESM3_ESM.docx]

Re: "Antibacterial Activity of Crocin-Loaded Niosomes against Foodborne Pathogens Isolated from Cream Pastries"

The corresponding author has been asked to revise the above submission on which you are listed as a contributing author. Meanwhile, we would be grateful if you would carefully check the author details, including spelling and sequence of given and family names; email and affiliation:

Corresponding author:
Masoomeh Amini
[dr_m_amini81@yahoo.com/](mailto:dr_m_amini81@yahoo.com/) amini.m@sina.tums.ac.ir
Department of Pathobiology, School of Public Health, Tehran University of Medical Sciences, IR

Ebrahim Molaee-aghaee
emolaeeaghaee@tums.ac.ir
Division of Food Safety and Hygiene, Department of Environmental Health Engineering, School of Public Health, Tehran University of Medical Sciences IR

Ramin Akbari Dehcheshmeh
em.aqaee@gmail.com
Department of Pathobiology, School of Public Health, Tehran University of Medical Sciences, IR

Razieh Noroozi
Raznoroozi@yahoo.com
Division of Food Safety and Hygiene, Department of Environmental Health Engineering, School of Public Health, Tehran University of Medical Sciences IR

Mohammad Reza Pourmand
pourmand@gmail.com
Department of Pathobiology, School of Public Health, Tehran University of Medical Sciences, IR

Parsa Abassi
parsa.abbasi98@gmail.com
Department of Medical Nanotechnology, School of Advanced Technologies in Medicine, Tehran University of Medical Sciences, IR

Samira Karimaei
samiraka796@gmail.com
Department of Pathobiology, School of Public Health, Tehran University of Medical Sciences, IR

If you spot any errors, please contact the corresponding author directly to ensure the revision moves smoothly through the submission process.

Kind regards,

Editorial Assistant
BMC Microbiology
